# Efficacy and Safety of Bilastine in the Treatment of Allergic Rhinitis: A Systematic Review and Meta-analysis

**DOI:** 10.3389/fphar.2021.731201

**Published:** 2022-01-10

**Authors:** Aranjit Singh Randhawa, Norhayati Mohd Noor, Mohd Khairi Md Daud, Baharudin Abdullah

**Affiliations:** ^1^ Department of Otorhinolaryngology-Head & Neck Surgery, School of Medical Sciences, Universiti Sains Malaysia, Kubang Kerian, Malaysia; ^2^ Department of Family Medicine, School of Medical Sciences, Universiti Sains Malaysia, Kubang Kerian, Malaysia

**Keywords:** bilastine, oral antihistamine, allergic rhinitis, efficacy, adverse events

## Abstract

Bilastine is a non-sedating second generation H1 oral antihistamine (OAH) for treating allergic rhinitis (AR) patients. The effect of bilastine has not previously been evaluated in a meta-analysis. The aim of this review was to determine the efficacy and safety of bilastine in treating AR. An electronic literature search was performed using Cochrane Central Register of Controlled Trials (CENTRAL), MEDLINE, Science Direct and Google Scholar up to March 2021. Randomized controlled trials comparing bilastine with placebo and standard pharmacotherapy were included. The included studies must have diagnosis of AR established by clinicians and the outcomes must have a minimum of 2 weeks of follow-up period. The primary outcomes assessed were total symptom score (TSS), nasal symptom score (NSS) and non-nasal symptom score (NNSS). The secondary outcomes were discomfort due to rhinitis, quality of life (QOL) and adverse events. The risk of bias and quality of evidence for all studies were appraised. The meta-analysis was done using Review Manager 5.3 software based on the random-effects model. The search identified 135 records after removal of duplicates. Following screening and review of the records, fifteen full-text articles were assessed for eligibility. Five trials involving 3,329 patients met the inclusion criteria. Bilastine was superior to placebo in improving TSS, NSS, NNSS, rhinitis discomfort score and QOL but has comparable efficacy with other OAHs in TSS, NSS, NNS, rhinitis discomfort score and QOL. There was no difference in adverse effects when bilastine was compared against placebo and other OAHs except for somnolence. Bilastine has fewer incidence of somnolence compared to cetirizine. The overall quality of evidence ranged from moderate to high quality. Bilastine is effective and safe in treating the overall symptoms of AR with comparable efficacy and safety with other OAHs except somnolence. Whilst bilastine has similar efficacy to cetirizine, somnolence is notably less in bilastine.

## 1 Introduction

Rhinitis is described as an inflammation of the nasal epithelium causing rhinorrhoea, nasal blockage, itching and sneezing. The commonest form of rhinitis is allergic rhinitis (AR) with the occurrence of the rhinitis symptoms and allergic sensitization following exposure to allergens (Burbach et al., 2009; Braido et al., 2014). Most common aeroallergens involved include house dust mite, weed pollen, tree pollen, grass pollen, cat, dog and moulds (Burbach et al., 2009). Allergic Rhinitis and Its Impact on Asthma (ARIA) guidelines classified AR as seasonal (intermittent) or perennial (persistent) based on the duration of symptoms and graded its severity as mild, moderate to severe according to its impact on the quality of life (Bousquet et al., 2008). In allergic inflammation, histamine plays a critical role in the pathophysiologic process. In genetically predisposed individuals, the IgE type-specific antibodies are secreted post-exposure to an allergen which then link to the receptors on the surface of mast cells and basophils. This interaction causes exocytosis of histamine and different inflammatory mediators such as cytokines and platelet-activating factor. H1, H2, H3 and H4 are the four main histamine receptor subtypes, but the allergic response is mainly mediated by the H1 receptor subtype (Zazzali et al., 2012).

Bilastine, a novel non-sedating second-generation oral antihistamine (OAH) drug, is a H1 receptor inverse agonist and belongs to the piperidine class (Braido et al., 2014). It has high specific affinity for the H1-receptor and binds to various sites on the H1 receptor. Allergic inflammation is downregulated by binding of H1 antihistamines to H1 receptors which inhibits histamine activity on small blood vessels and sensory neurons (Church et al., 2013). Though it does not antagonize the binding of histamine directly, its affinity and binding to H1 receptor produces an opposite effect. The main difference between the second generation and the first generation OAH is the adverse effect of drowsiness (Bousquet et al., 2008). In second generation OAH there is less absorption *via* the blood brain barrier and thus, there is minimal penetration to the brain with less central nervous effects compared to the first generation OAH. To date, there is no meta-analysis evaluating the effect of bilastine as a pharmacological treatment for AR. This meta-analysis aims to determine its efficacy and safety in treating AR.

## 2 Materials and Methods

Our systematic review was done according to a protocol published in PROSPERO with identification serial number as CRD 42019125401. The methods and reporting were based on the Cochrane Collaboration and the preferred reporting items for systematic reviews and meta-analyses statement (Moher et al., 2009; Cumpston et al., 2019). The evaluation was done according to the Grading of Recommendations Assessment, Development, and Evaluation (GRADE) guideline (Guyatt et al., 2008).

### 2.1 Eligibility Criteria

Randomized control trials (RCTs) comparing bilastine with placebo or no treatment would be included. In addition, trials involving OAHs, intranasal corticosteroid nasal sprays, and leukotriene receptor antagonists would be included when available. All aged groups diagnosed with AR of either gender or any ethnicity were eligible. The included studies must have diagnosis of allergic rhinitis established by clinicians and the outcomes must have a minimum of 2 weeks of follow-up period.

### 2.2 Search Strategy

We searched the Cochrane Central Register of Controlled Trials (CENTRAL), MEDLINE, Science Direct and Google Scholar up to March 2021. The search strategy is shown in Supplementary Table S1. The search was restricted to English language only. We checked the reference list of identified RCTs and reviewed articles to find unpublished trials or trials not identified by electronic searches. We searched for ongoing trials through the World Health Organization http://www.who.int/ictrp/en/ and International Clinical Trials Registry Platform www.clinicaltrials.gov.

### 2.3 Study Selection

Two review authors (ASR, NMN) scanned the titles and abstracts independently from the searches and obtained full-text articles when they appeared to meet the eligibility criteria or when there was insufficient information to assess eligibility. We independently assessed the eligibility of the trials and documented the reasons for exclusion. We resolved any disagreements between the review authors by discussion. We contacted the trial authors if clarification was needed.

### 2.4 Data Extraction

Using data extraction form, we extracted study setting, participant characteristics (age, gender, ethnicity), methodology (number of participants randomized and analyzed, duration of follow-up), method used for diagnosing AR and classifying perennial or seasonal type of AR from each of the selected trials. The primary outcomes assessed were the total symptom score (TSS), nasal symptom score (NSS) and non-nasal symptom score (NNSS). The secondary outcomes were discomfort due to rhinitis, quality of life (QOL) and adverse events (AE) such as headache, somnolence or fatigue. We resolved any disagreements by discussion.

### 2.5 Risk of Bias Assessment

Assessment of risk of bias for all studies was performed based on the Cochrane Handbook (Cumpston et al., 2019). It was classified into low risk, unclear risk or high risk based on random sequence generation, allocation concealment, blinding of participants and personnel, blinding of outcome assessors, completeness of outcome data, the selectivity of outcome reporting and other bias. Any disagreements were resolved by discussion.

### 2.6 Grading Quality of Evidence

We used the principles of the Grades of Recommendation, Assessment, Development and Evaluation (GRADE) approach for evaluating the quality of evidence in systematic reviews (Guyatt et al., 2008). The four levels of quality are very low, low, moderate, or high, depending on the risk of bias, inconsistency, indirectness, imprecision and publication bias. The quality of evidence for each outcome was incorporated as the “Summary of Findings” (SoF) table.

### 2.7 Statistical Analysis

The data analysis was performed by using Review Manager 5.3 software based on the random-effects model. Heterogeneity was interpreted as follows: 0–40% might not be important; 30–60% may represent moderate heterogeneity; 50–90% may represent substantial heterogeneity, and 75–100% would be considerable heterogeneity (Cumpston et al., 2019). We explored the potential sources of heterogeneity when significant heterogeneity was present. Based on data availability, the treatment measurement for continuous outcomes was accomplished using mean differences (MDs) or standard mean difference (SMD) and relative risk (RR) with 95 % confidence intervals (CIs). The subgroup analyses included the duration of treatment and age of children (above two and below 12 years old). The unit of analysis errors were checked for the included trials.

## 3 Results

### 3.1 Study Selection

**FIGURE 1:**
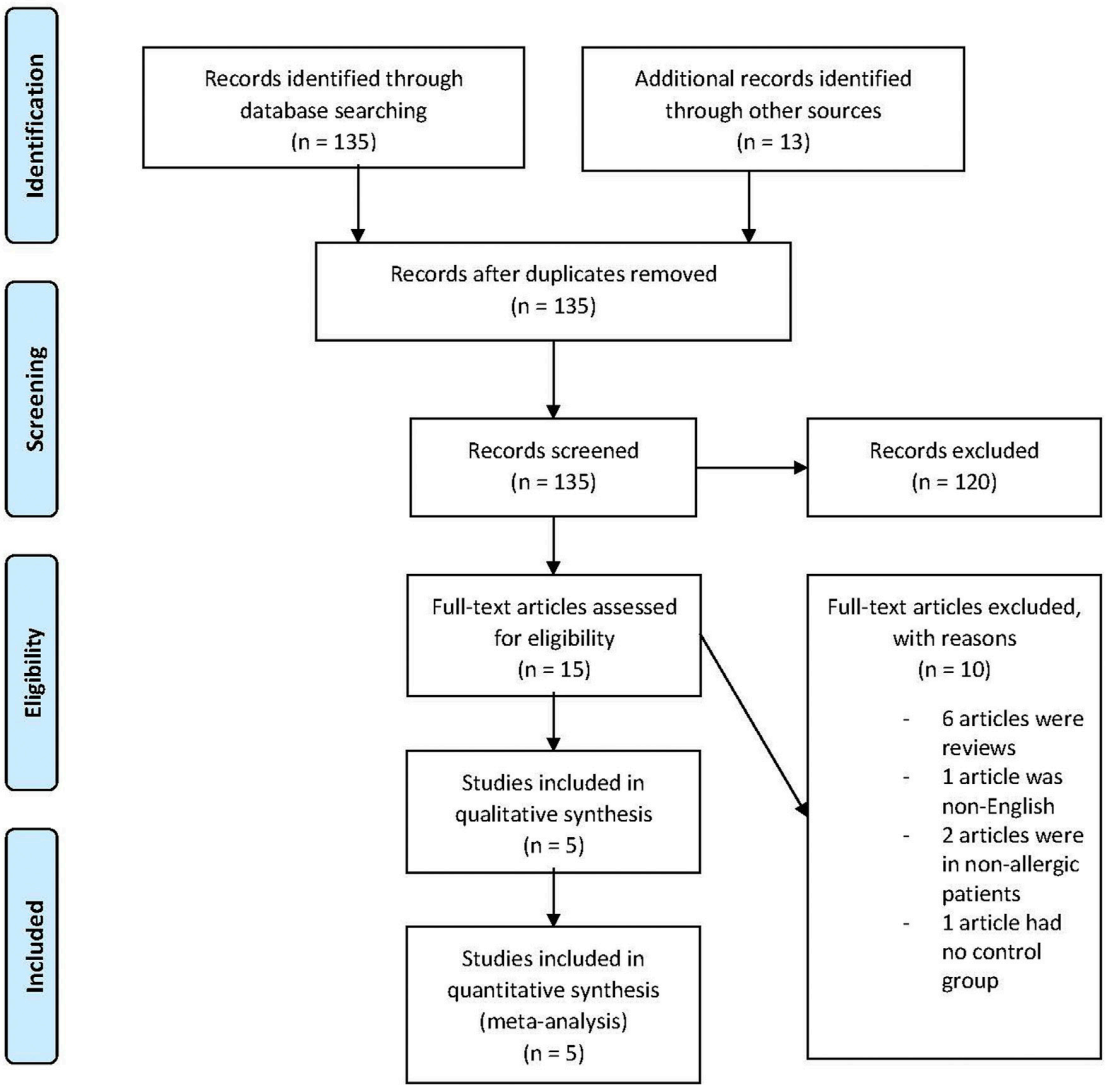
Flow diagram of study selection.

We retrieved 135 records from the search of the electronic databases and additional 13 records from other sources. Following removal of the duplicates, 135 records left. After screening, 87 records were excluded. After reviewing 48 records possibly meeting the inclusion criteria, 33 records were excluded. Fifteen full-text articles were assessed for eligibility and 10 records were excluded. Among the excluded articles were six review articles (Kruszewski, 2009; Braido et al., 2014; Kowal and DuBuske, 2014; Ridolo et al., 2015; Wang et al., 2016; Pasko et al., 2017), one article (Kruszewski, 2009) published in non-English medium, two trials performed in non-allergic rhinitis patients (Valk et al., 2016; Remenyi et al., 2018) and one trial (Demonte et al., 2018) had no control group. Hence, five trials were included for qualitative synthesis and meta-analysis (Figure 1). All the trials involved bilastine versus placebo and other OAHs. There were no trials comparing bilastine against any intranasal corticosteroid nasal sprays and leukotriene receptor antagonists.

### 3.2 Participants

Five trials (Bachert et al., 2009; Kuna et al., 2009; Sastre et al., 2012; Novak et al., 2016; Okubo et al., 2017) of 3,329 participants met the inclusion criteria. The characteristics of the included trials are shown in Table 1. All five trials (Bachert et al., 2009; Kuna et al., 2009; Sastre et al., 2012; Novak et al., 2016; Okubo et al., 2017) were conducted as multicenter trials. Four of the five trials (Bachert et al., 2009; Kuna et al., 2009; Sastre et al., 2012; Okubo et al., 2017) were conducted in the population with age group 12–74 years old and one trial (Novak et al., 2016) was conducted in the population with age group 2–12 years old. All five trials (Bachert et al., 2009; Kuna et al., 2009; Sastre et al., 2012; Novak et al., 2016; Okubo et al., 2017) confirmed AR by positive skin prick test or positive specific IgE test. Two trials (Bachert et al., 2009; Kuna et al., 2009) enrolled patients with documented clinical history of seasonal AR to a pollen allergen for at least 2 years. Two trials (Sastre et al., 2012; Okubo et al., 2017) consist of patients who have a minimum 2-years history of symptoms indicative of perennial AR. Two trials (Bachert et al., 2009; Kuna et al., 2009) reported the inclusion of participants with a baseline 12-h reflective nasal symptom score ≥36 assessed on the last 3 days during the screening period.

**TABLE 1:** The characteristics of included studies.

Study reference	Age (years)	Number of participants	Subject	Indication	Comparators	Outcome measures
Bachert et al. (2009)	12–70	721	SAR	At least 2-years history and positive skin prick test (common grass pollen and tree pollen, including perennial allergens	Desloratadine, placebo	TSS, NSS, NNSS, QOL, AE
Kuna et al. (2009)	12–70	683	SAR	At least 2-years history and positive skin prick test (season pollen allergens) or specific IgE to at least one seasonal allergen	Cetirizine, placebo	TSS, NSS, NNSS, AE
Novak et al. (2016)	2–12	509	Allergic rhinoconjunctivitis/ chronic urticaria	Positive skin prick test or specific IgE	Placebo	AE
Okubo et al. (2017)	18–74	765	PAR	At least 2-years history and positive nasal provocation test (house dust disc) or specific IgE (at least one house dust mite)	Placebo, fexofenadine	TSS, QOL,AE
Sastre et al. (2011)	12–70	651	PAR	At least 2-years history and positive skin prick test (house dust mites, cockroaches, molds or animal danders	Placebo, cetirizine	TSS, AE

*PAR*, perennial allergic rhinitis; *SAR,* seasonal allergic rhinitis; *TSS*, total symptom score; *NSS*, nasal symptom score; *NNSS*, non-nasal symptom score; *QOL*, quality of life; *AE*, adverse events.

All five trials (Bachert et al., 2009; Kuna et al., 2009; Sastre et al., 2012; Novak et al., 2016; Okubo et al., 2017) reported exclusion of participants due to active infections such as nasal septal ulcers or polyps, asthma, as well as any other nasal, ocular, or ear disorders that could interfere with efficacy evaluation, as well as viral conjunctivitis, otitis media, sinusitis, nasal polyps, repetitive nasal hemorrhage, and any previous history of intranasal surgery. Patients who were hypersensitive to H1 antihistamine and benzimidazoles and those taking specific H1 or H2 antihistamines within 3 days to 6 weeks, systemic or intranasal corticosteroids within 4 weeks, and intranasal and systemic decongestants within 3 days of randomization to treatment were excluded from the study. Patients who had received immunotherapy within 24 h before the study visit or central nervous system acting agents were also excluded.

### 3.3 Intervention

Participants in the trials were randomized into intervention and control groups. The intervention was bilastine 20 mg daily in four trials (Bachert et al., 2009; Kuna et al., 2009; Sastre et al., 2012; Okubo et al., 2017) while one trial (Novak et al., 2016) administered bilastine 10 mg daily. All five trials (Bachert et al., 2009; Kuna et al., 2009; Sastre et al., 2012; Novak et al., 2016; Okubo et al., 2017) had placebo for comparison. Two trials (Kuna et al., 2009; Sastre et al., 2012) compared bilastine to cetirizine as a second comparator. One trial each had desloratadine (Bachert et al., 2009) and fexofenadine (Okubo et al., 2017) as comparators. Bilastine 20 mg was administered orally in tablet form in four trials (Bachert et al., 2009; Kuna et al., 2009; Sastre et al., 2012; Okubo et al., 2017) and bilastine 10 mg as an oral dispersible tablet (either swallowed or dissolved in water) in one trial (Novak et al., 2016). Bilastine 10 mg was administered once daily in the morning 1 hour before breakfast or 2 hours after breakfast for 12 weeks in one trial (Novak et al., 2016), bilastine 20 mg for 14 days in three trials (Bachert et al., 2009; Kuna et al., 2009; Okubo et al., 2017) and 28 days in one trial (Sastre et al., 2012). Two trials (Kuna et al., 2009; Sastre et al., 2012) administered tablet cetirizine 10 mg daily. Cetirizine 10 mg was given once daily and an hour before or 2 hours after breakfast. One trial (Bachert et al., 2009) administered tablet desloratadine 5 mg once daily one to 2 hours before breakfast. One trial (Okubo et al., 2017) dispensed tablet fexofenadine 60 mg given twice daily an hour before or 2 hours after breakfast. Two trials (Bachert et al., 2009; Kuna et al., 2009) reported follow-ups at day 7 and 14 of treatment, one trial (Novak et al., 2016) at week 4, 8, and 12 of treatment and week 16 post-treatment, one trial (Sastre et al., 2012) at day 14 and 28 of treatment and one trial (Okubo et al., 2017) had follow-up 4–7 days following the end of 14 days of treatment.

### 3.4 Outcomes

A total of four trials (Bachert et al., 2009; Kuna et al., 2009; Sastre et al., 2012; Okubo et al., 2017) reported the primary outcomes. The total symptom score reported was the sum of four nasal symptoms (congestion, rhinorrhea, itching and sneezing) and six non-nasal symptoms (ocular itching, burning, a sensation of foreign body in the eye, tearing, redness and itching of ears or palate). Four trials (Bachert et al., 2009; Kuna et al., 2009; Sastre et al., 2012; Okubo et al., 2017) reported that they had measured the participants for the total symptom score. Out of these four, three trials (Bachert et al., 2009; Kuna et al., 2009; Sastre et al., 2012) reported evaluating the severity of the nasal and ocular symptoms on a four-point Likert scale, while one trial (Okubo et al., 2017) evaluated nasal symptoms which were sneezing, rhinorrhea or nasal congestion using five-point scale. One trial (Bachert et al., 2009) included four nasal and six non-nasal symptoms for the total symptom score. One trial (Kuna et al., 2009) reported four nasal and three non-nasal symptoms (ocular itching, redness and tearing) for the total symptom score. One trial (Okubo et al., 2017) included four nasal symptoms for the total symptom score and one trial (Sastre et al., 2012) included four nasal and two non-nasal symptoms (ocular redness and tearing) for the total symptom score. As the continuous outcome variables were expressed in different scales for the total symptom score, we estimated the SMD. All four trials (Bachert et al., 2009; Kuna et al., 2009; Sastre et al., 2012; Okubo et al., 2017) met our pre-specified duration of follow-up for at least 2 weeks. One trial (Sastre et al., 2012) was conducted for 28 days with visits after day 14 and day 28 of treatment. Two trials (Bachert et al., 2009; Kuna et al., 2009) reported nasal symptom score and non-nasal symptom score. Secondary outcomes were reported in all five trials (Bachert et al., 2009; Kuna et al., 2009; Sastre et al., 2012; Novak et al., 2016; Okubo et al., 2017). Two trials (Bachert et al., 2009; Kuna et al., 2009) assessed discomfort due to rhinitis at day 0, 7 and 14 on a 0–100 mm visual analogue scale (VAS), ranging from 0 for no discomfort to 100 for extreme discomfort. Two trials (Bachert et al., 2009; Okubo et al., 2017) reported the quality of life of patients. One trial (Bachert et al., 2009) assessed the quality of life using the Rhinoconjunctivitis Quality of Life Questionnaire (RQLQ), which evaluates the effect of symptoms and treatment. By means of RQLQ, the patients scored each item on a seven-point scale. The higher the score, the poorer the quality of life. One trial (Okubo et al., 2017) reported quality of life using the Japanese Allergic Rhinitis Standard Questionnaire. The quality of life-related questionnaire included 17 items concerning reduced productivity at work/home/school, poor mental concentration, reduced thinking power, impaired reading book/paper, reduced memory loss, limitation of outdoor life (e.g., sports, picnic), limitation of going out, hesitation visiting friend or relatives, reduced contact with friends or others by telephone or conversation, not an easy person to be around, impaired sleeping, tiredness, fatigue, frustration, irritability, depression; and unhappiness. Each item was evaluated on a five-point scale as 0 (no significant problem),1 (mild problem), 2 (moderately severe), 3 (severe) and 4 (very severe). The higher the score, the poorer the quality of life.

### 3.5 Risk of Bias and Quality of Studies

**FIGURE 2:**
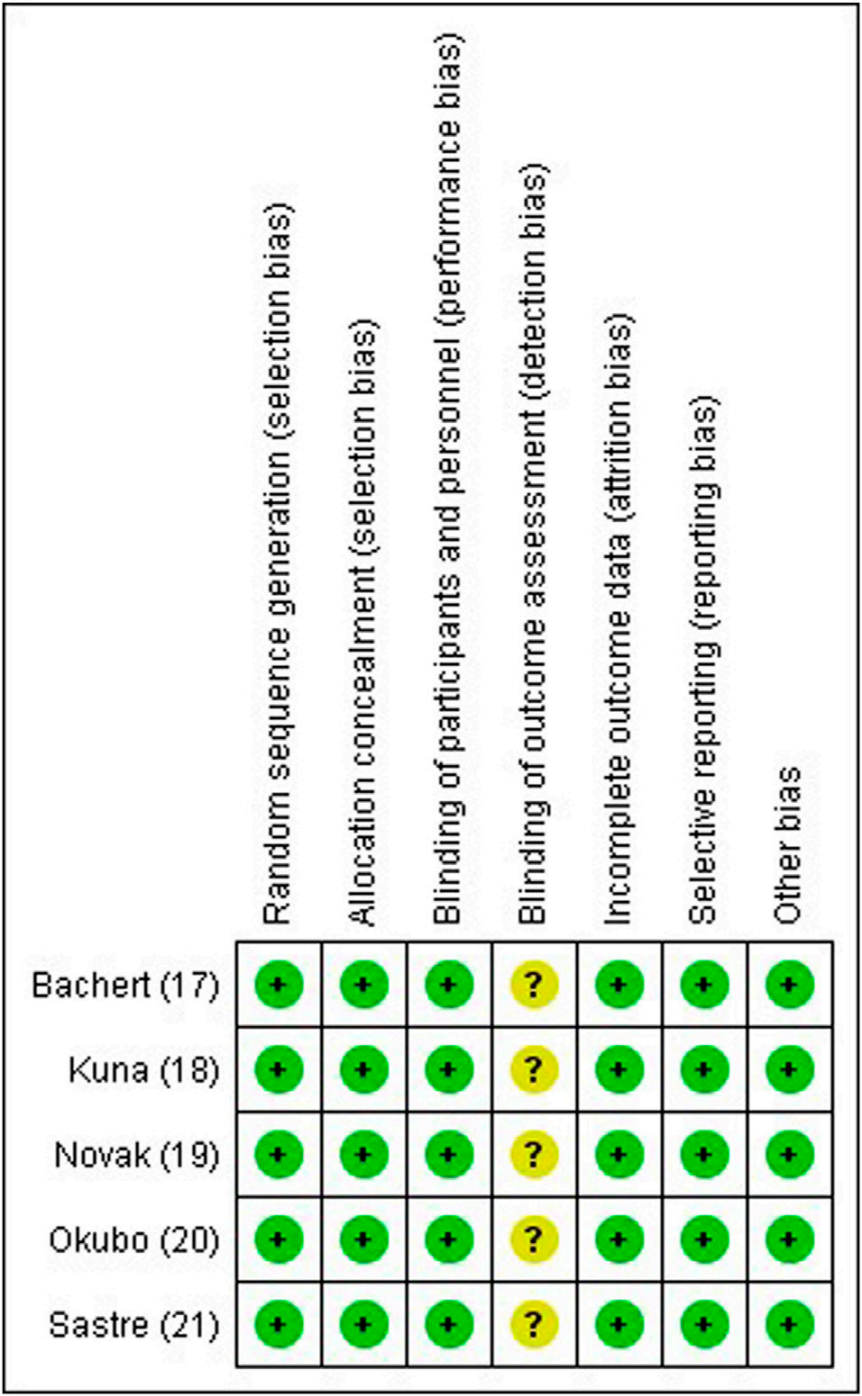
Risk of bias summary.

All studies had low risk in terms of random sequence generation, allocation concealment, blinding of participants and personnel, incomplete outcome data and selective reporting. There was unclear risk for blinding of outcome assessment for all five studies. There was no other bias detected. The authors judgement for each risk of bias item for each study is shown in Figure 2. The GRADE assessment indicated the overall quality of evidence ranged from moderate to high quality, implying that the estimated effect is probably close or similar to the actual effect.

### 3.6 Comparisons and Effects of Interventions

Four comparisons were assessed in this review. The primary and secondary outcomes were evaluated for each comparison. The four comparisons were bilastine versus placebo, bilastine versus cetirizine, bilastine versus desloratadine and bilastine versus fexofenadine.

#### 3.6.1 Total Symptom Score

**FIGURE 3:**
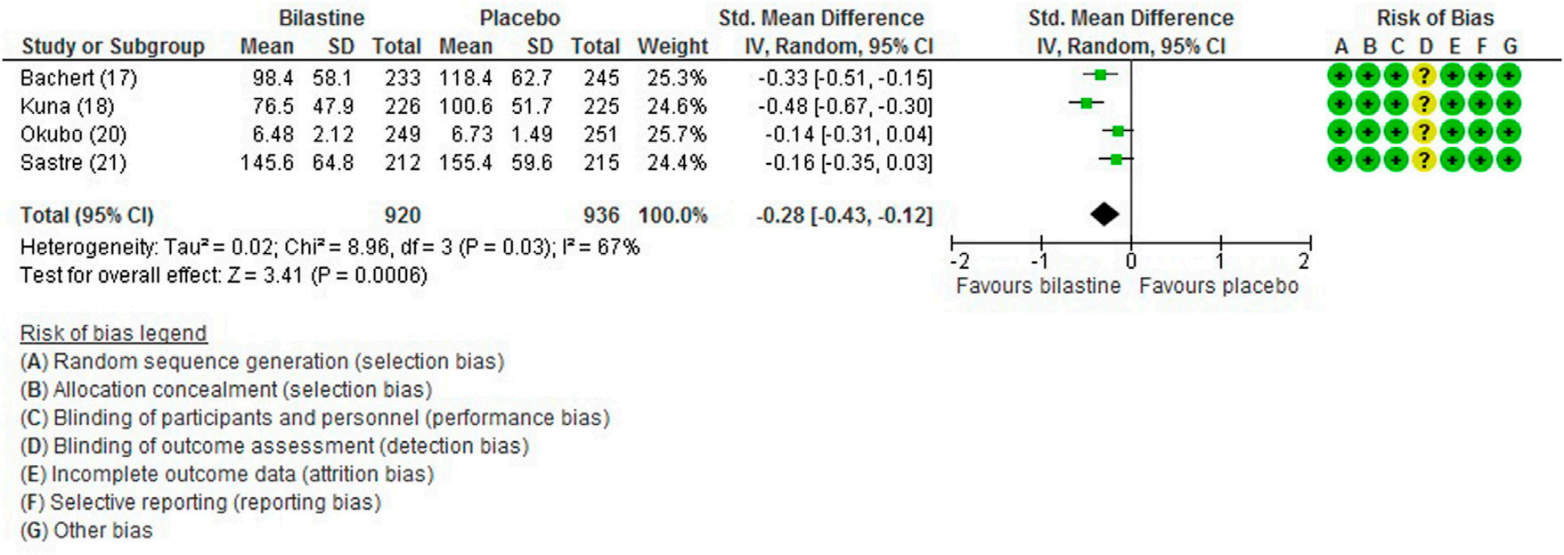
Comparison between bilastine and placebo for total symptom score. *Std* Standard, *SD* Standard deviation, *IV* inverse variance, *CI* Confidence interval, *df* degrees of freedom.

**FIGURE 4:**
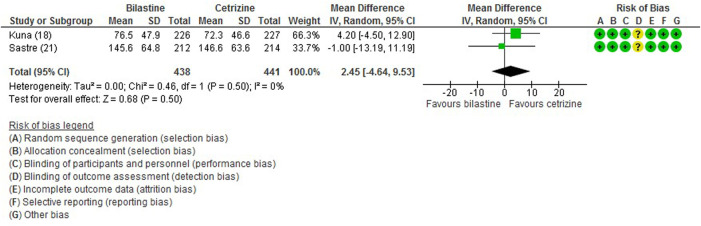
Comparison between bilastine and cetirizine for total symptom score. *SD* Standard deviation, *IV* inverse variance, *CI* Confidence interval, *df* degrees of freedom.

For comparison between bilastine and placebo, there were four trials (Bachert et al., 2009; Kuna et al., 2009; Sastre et al., 2012; Okubo et al., 2017) that reported the outcome of the total symptom score. When compared against placebo, there was a significant reduction in the bilastine treated group (four trials, 1856 participants; SMD −0.28, 95% CI −0.43 to −0.12; *p* < 0.001; I^2^ = 67%; moderate quality of evidence) (Figure 3; Table 2). There was no difference between bilastine and cetirizine for the total symptom score (Kuna et al., 2009; Sastre et al., 2012) (two trials, 879 participants; MD 2.45, 95% CI -4.64 to 9.53; *p* = 0.50; I^2^ = 0%; high quality of evidence) (Figure 4; Table 3). There was no difference between bilastine and desloratadine for the total symptom score (Bachert et al., 2009) (475 participants; MD −2.10, 95% CI −12.25 to 8.05). There was no difference between bilastine and fexofenadine for the total symptom score (Okubo et al., 2017) (496 participants; MD 0.06, 95% CI −0.40–0.42).

**TABLE 2:** Summary of findings for bilastine versus placebo.

Bilastine versus placebo for allergic rhinitis					
Patient or population: Allergic rhinitis, intervention: Bilastine, comparison: Placebo					
Outcomes	Anticipated absolute effects* (95% CI)		**Relative effect(95% CI)**	**№ of participants(studies)**	**Certainty of the evidence (GRADE)**
	Risk with placebo	Risk with bilastine			
Total nasal symptom	The mean total nasal symptom was 0	SMD 0.28 lower(0.43 lower to 0.12 lower)	—	1856(4 RCTs)	⊕⊕⊕⊝MODERATE ^a^
Nasal symptom	The mean nasal symptom was 0	MD 12 lower(17.78 lower to 6.22 lower)	—	929(2 RCTs)	⊕⊕⊕⊝MODERATE ^b^
Non-nasal symptom	The mean non-nasal symptom was 0	MD 9.80 lower (13.27 lower to 6.33 lower)	—	929(2 RCTs)	⊕⊕⊕⊕HIGH
Discomfort due to rhinitis	The mean discomfort due to rhinitis was 0	MD 15.39 lower (22.74 lower to 8.04 lower)	—	929(2 RCTs)	⊕⊕⊕⊝MODERATE ^c^
Headache	Study population		RR 1.08 (0.72–1.64)	2,373 (5 RCTs)	⊕⊕⊕⊝MODERATE ^d^
	111 per 1,000	119 per 1,000 (80–181)			
Somnolence	Study population		RR 1.15 (0.63–2.07)	1864 (4 RCTs)	⊕⊕⊕⊕HIGH
	21 per 1,000	25 per 1,000 (13–44)			
Fatigue	Study population		RR 0.84 (0.23–3.02)	1,364 (3 RCTs)	⊕⊕⊕⊝MODERATE ^e^
	23 per 1,000	19 per 1,000 (5–70)			

*The risk in the intervention group (and its 95% confidence interval) is based on the assumed risk in the comparison group and the relative effect of the intervention (and its 95% CI).CI: Confidence interval; RR: Risk ratio; SMD: Standardized mean difference; MD: Mean difference.

a The heterogeneity is 67%.

b The heterogeneity is 50%.

c The heterogeneity is 57%.

d The heterogeneity is 64%.

e The heterogeneity is 56%.

*RCT*, randomized controlled trial.

**TABLE 3:** Summary of findings for bilastine versus cetirizine.

Bilastine versus cetirizine for allergic rhinitis					
Patient or population: Allergic rhinitisIntervention: bilastineComparison: Cetirizine					
Outcomes	Anticipated absolute effects^*^ (95% CI)		Relative effect (95% CI)	№ of participants (studies)	Certainty of the evidence (GRADE)
	**Risk with placebo**	**Risk with bilastine**			
Total nasal symptom	The mean total nasal symptom was 0	MD 2.45 higher (4.64 lower to 9.53 higher)	—	879 (2 RCTs)	⊕⊕⊕⊕HIGH
Headache	Study population		RR 1.58(0.96–2.60)	886 (2 RCTs)	⊕⊕⊕⊕HIGH
	67 per 1,000	107 per 1,000(69–165)			
Somnolence	Study population		RR 0.38(0.17–0.86)	886 (2 RCTs)	⊕⊕⊕⊕HIGH
	72 per 1,000	27 per 1,000(14–52)			
Fatigue	Study population		RR 0.56(0.02–18.98)	886 (2 RCTs)	⊕⊕⊕⊝MODERATE ^a^
	29 per 1,000	16 per 1,000(1–554)			

*The risk in the intervention group (and its 95% confidence interval) is based on the assumed risk in the comparison group and the relative effect of the intervention (and its 95% CI).CI: Confidence interval; RR: Risk ratio; MD: Mean difference.

a The heterogeneity is 87%.

*RCT*, randomized controlled trial.

#### 3.6.2 Nasal Symptom Score

**FIGURE 5:**
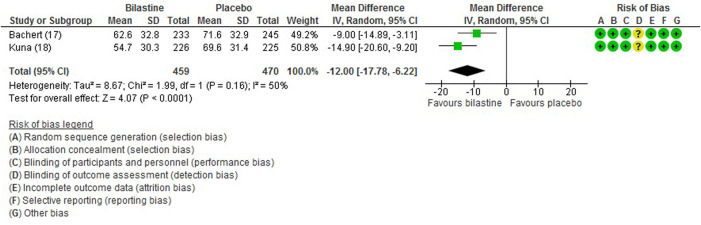
Comparison between bilastine and placebo for nasal symptom score. *SD* Standard deviation, *IV* inverse variance, *CI* Confidence interval, *df* degrees of freedom.

For comparison between bilastine and placebo, there were two trials (Bachert et al., 2009; Kuna et al., 2009) that reported the outcome of the nasal symptom score. Bilastine showed a significant reduction in the nasal symptom score compared with placebo (two trials, 929 participants; MD −12.00, 95% CI −17.78 to −6.22; *p* < 0.001; I^2^ = 50%; moderate quality of evidence) (Figure 5; Table 2). There was no difference between bilastine and desloratadine for the nasal symptom score (Bachert et al., 2009) (475 participants; MD −1.20, 95% CI −6.83 to 4.43). Likewise, there was no difference between bilastine and cetirizine for the nasal symptom score (Kuna et al., 2009) (453 participants; MD 4.60, 95% CI −0.81–10.01).

#### 3.6.3 Non-nasal Symptom Score

**FIGURE 6:**
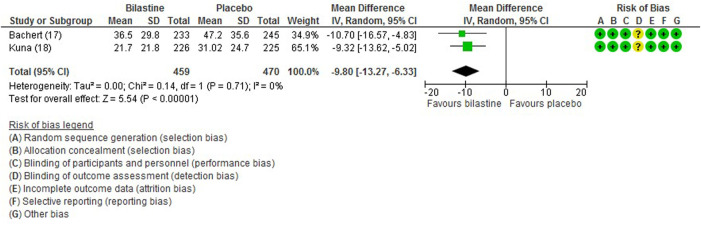
Comparison between bilastine and placebo for non-nasal symptom score. *SD* Standard deviation, *IV* inverse variance, *CI* Confidence interval, *df* degrees of freedom.

For comparison between bilastine and placebo, there were two trials (Bachert et al., 2009; Kuna et al., 2009) that reported the outcome of the non-nasal symptom score. Bilastine was significantly more effective than placebo in improving the non-nasal symptom score (two trials, 929 participants; MD -9.80, 95% CI −13.27 to −6.33; *p* < 0.001; I^2^ = 0%; high quality of evidence) (Figure 6; Table 2). There was no difference between bilastine and desloratadine for the non-nasal symptom score (Bachert et al., 2009) (475 participants; MD −0.72, 95% CI −6.17 to 4.73). There was no difference between bilastine and cetirizine for the non-nasal symptom score (Kuna et al., 2009) (453 participants; MD 4.60, 95% CI −0.81–10.01).

#### 3.6.4 Rhinitis-Associated Discomfort Score

**FIGURE 7:**
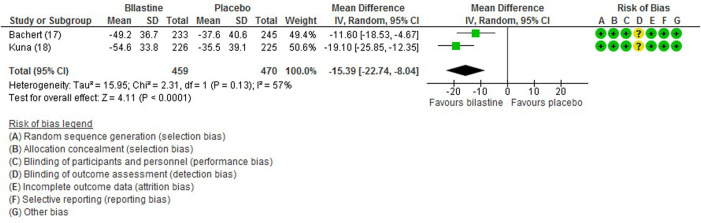
Comparison between bilastine and placebo for discomfort due to rhinitis. *SD* Standard deviation, *IV* inverse variance, *CI* Confidence interval, *df* degrees of freedom.

For comparison between bilastine and placebo, two trials (Bachert et al., 2009; Kuna et al., 2009) reported rhinitis-associated discomfort score. Bilastine showed decreased mean rhinitis discomfort score compared with placebo (two trials, 929 participants; MD −15.39, 95% CI −22.74 to −8.04; *p* < 0.001; I^2^ = 57%; moderate quality of evidence) (Figure 7; Table 2). There was no difference between bilastine and desloratadine for discomfort due to rhinitis score (Bachert et al., 2009) (475 participants; MD −0.50, 95% CI −7.17 to 6.17) and no difference between bilastine and cetirizine for discomfort due to rhinitis score (Kuna et al., 2009) (453 participants; MD 0, 95% CI −6.31 to 6.31).

#### 3.6.5 Quality of Life

One trial (Okubo et al., 2017) stated no difference in the quality of life between bilastine and placebo but the data was not reported. Another trial (Bachert et al., 2009) reported bilastine improved the quality of life of participants compared to placebo (478 participants; MD 0.30, 95% CI 0.02–0.58). Comparison of bilastine with desloratadine demonstrated no difference in the quality of life of participants (Bachert et al., 2009) (475 participants; MD 0.00, 95% CI −0.22 to 0.22).

#### 3.6.6 Adverse Events

**FIGURE 8:**
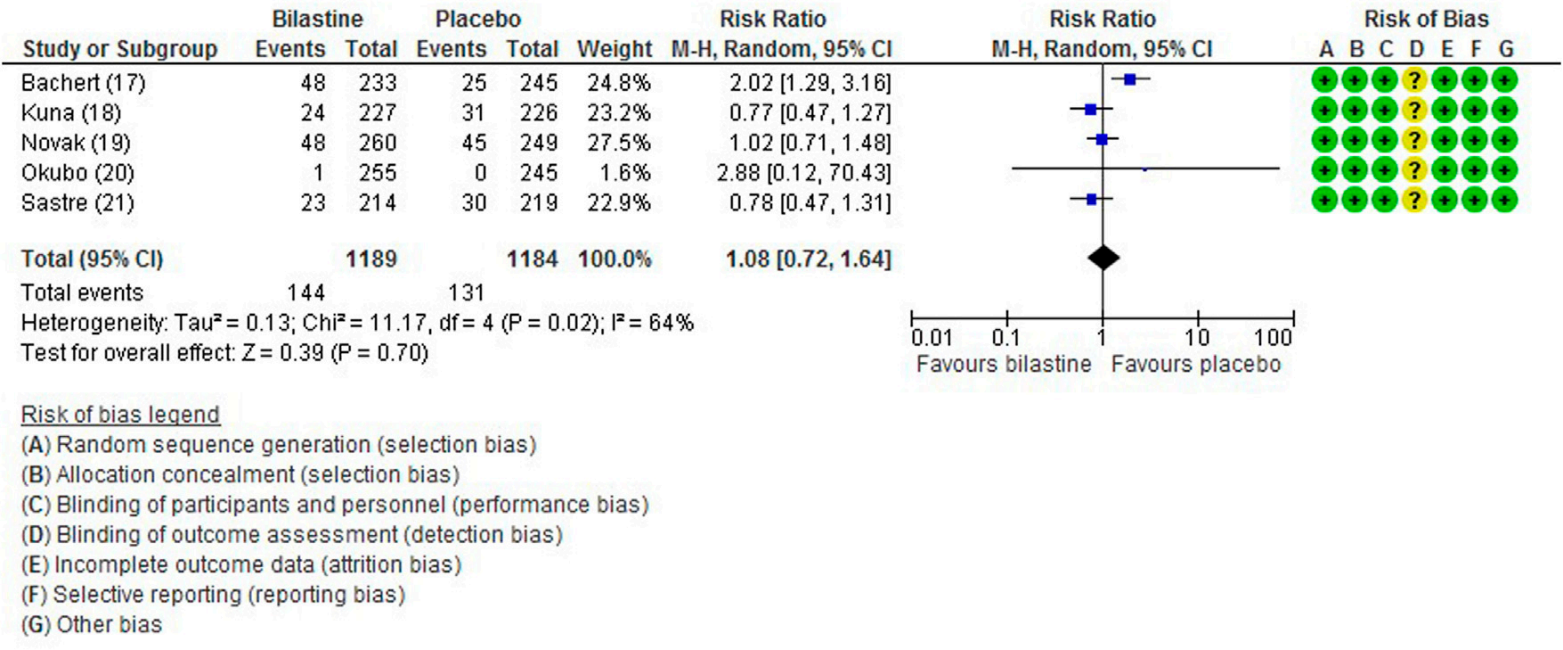
Comparison between bilastine and placebo for headache. *CI* Confidence interval, *df* degrees of freedom.

**FIGURE 9:**
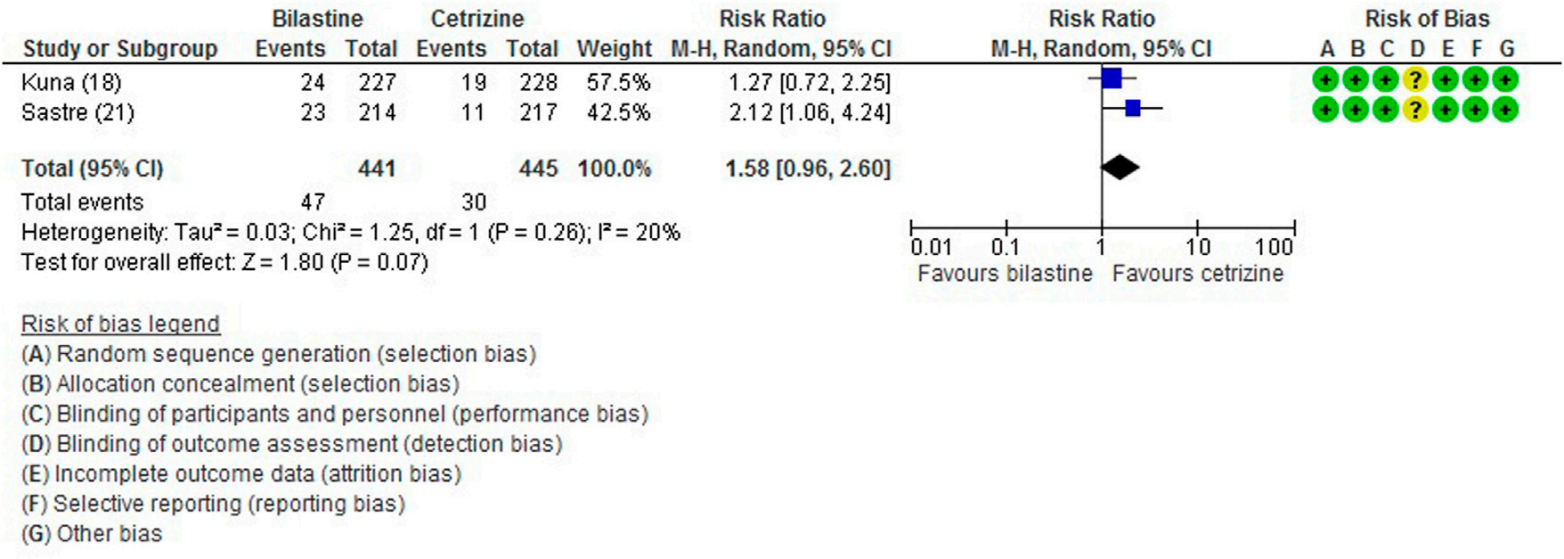
Comparison between bilastine and cetirizine for headache. *CI* Confidence interval, *df* degrees of freedom.

**FIGURE 10:**
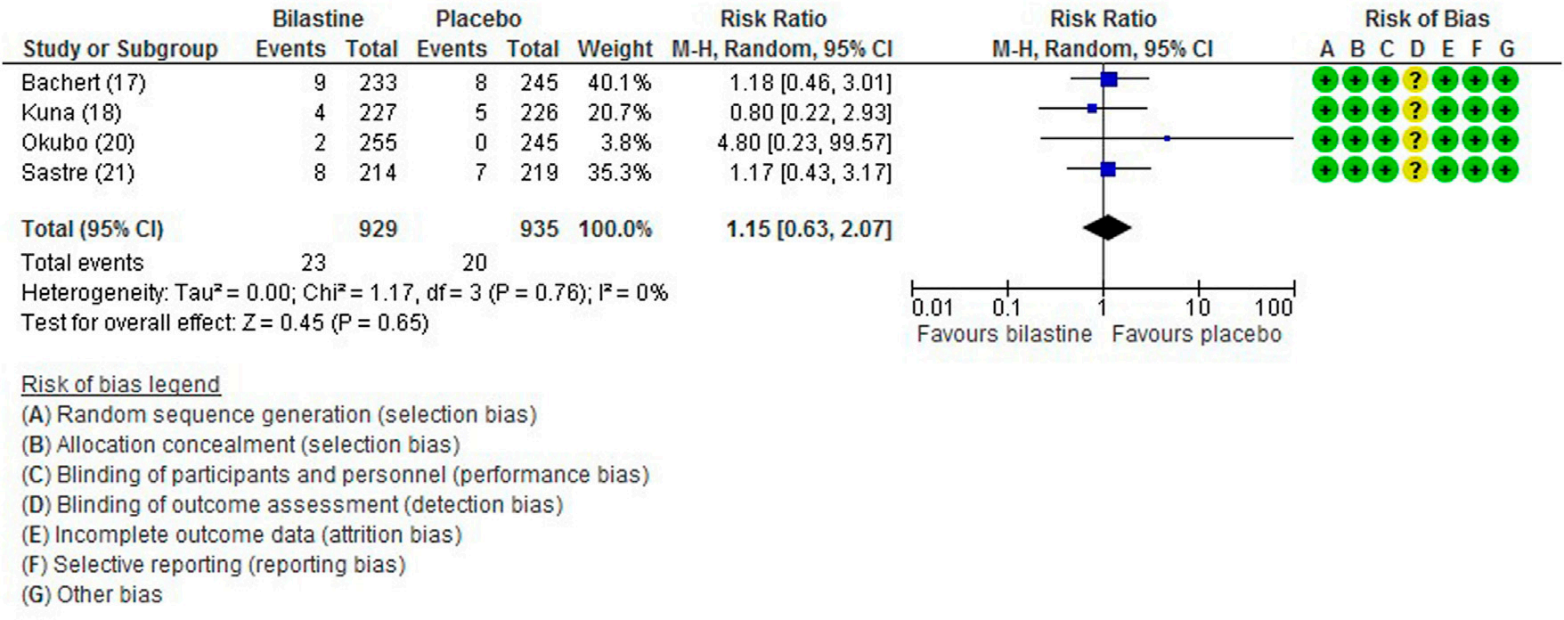
Comparison between bilastine and placebo for somnolence. *CI* Confidence interval, *df* degrees of freedom.

**FIGURE 11:**
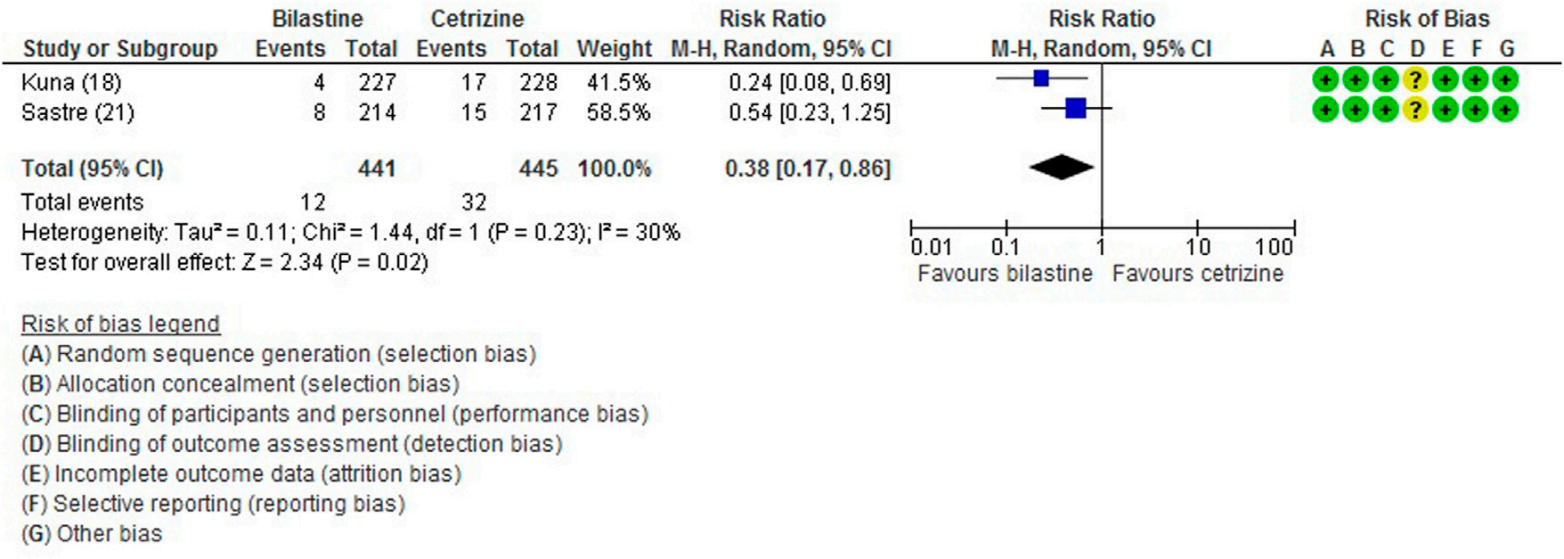
Comparison between bilastine and cetirizine for somnolence. *CI* Confidence interval, *df* degrees of freedom.

All five trials (Bachert et al., 2009; Kuna et al., 2009; Sastre et al., 2012; Novak et al., 2016; Okubo et al., 2017) reported adverse events. The most common adverse events were headache, somnolence, and fatigue. When compared to placebo, the frequency of headache was no difference to bilastine (Bachert et al., 2009; Kuna et al., 2009; Sastre et al., 2012; Novak et al., 2016; Okubo et al., 2017) (five trials, 2,373 participants; RR 1.08, 95% CI 0.72 to 1.64; *p* = 0.70; I^2^ = 64%; moderate quality of evidence) (Figure 8; Table 2). There was no difference between bilastine and cetirizine in the incidence of headache (Kuna et al., 2009; Sastre et al., 2012) (two trials, 886 participants; RR 1.58, 95% CI 0.96 to 2.60; *p* = 0.07; I^2^ = 20%; high quality of evidence) (Figure 9; Table 3). One trial each reported no difference in the incidence of headache between bilastine and desloratadine (Bachert et al., 2009) (475 participants; RR 1.00, 95% CI 0.60–1.66) and between bilastine and fexofenadine (Okubo et al., 2017) (496 participants; RR 0.50, 95% CI 0.05–5.46). Four trials reported no difference in somnolence between placebo and bilastine (Bachert et al., 2009; Kuna et al., 2009; Sastre et al., 2012; Okubo et al., 2017) (four trials, 1864 participants; RR 1.15, 95% CI 0.63 to 2.07; *p* = 0.65; I^2^ = 0%; high quality of evidence) (Figure 10; Table 2). Bilastine has fewer incidence of somnolence than cetirizine (Kuna et al., 2009; Sastre et al., 2012) (two trials, 886 participants; RR 0.38, 95% CI 0.17 to 0.86; *p* = 0.02; I^2^ = 30%; high quality of evidence) (Figure 11; Table 3). One trial each showed no difference in somnolence between bilastine and desloratadine (Bachert et al., 2009) (475 participants; RR 1.04, 95% CI 0.42–2.57) and between bilastine and fexofenadine (Okubo et al., 2017) (496 participants; RR 1.99, 95% CI 0.18–21.83). Three trials (Bachert et al., 2009; Kuna et al., 2009; Sastre et al., 2012) reported no difference in manifesting fatigue between bilastine and placebo (three trials, 1,364 participants; RR 0.84, 95% CI 0.23 to 3.02; *p* = 0.79; I^2^ = 56%; moderate quality of evidence) (Supplementary Figure S1, Table 2). Bilastine demonstrated no difference in the event of fatigue when compared to cetirizine (Kuna et al., 2009; Sastre et al., 2012) (two trials, 886 participants; RR 0.56, 95% CI 0.02 to 18.98; *p* = 0.75; I^2^ = 87%; moderate quality of evidence) (Supplementary Figure S2, Table 3). One trial (Bachert et al., 2009) reported no difference between bilastine and desloratadine in the event of fatigue (475 participants; RR 2.08, 95% CI 0.53–8.21). There was no serious adverse event or death reported by all 5 trials.

## 4 Discussion

### 4.1 Summary of Main Findings

To the best of our knowledge, this is the first meta-analysis to assess the efficacy and safety of bilastine in the treatment of allergic rhinitis. It includes a total of five randomized controlled trials that met the inclusion criteria. We performed a thorough and extensive literature review to assess the effectiveness and safety of bilastine in the treatment of AR compared with placebo and standard pharmacological treatments. The diagnosis of AR was objectively made, and we were certain that we included all studies that investigated AR. We were able to determine the common side effects from the reported incidence of adverse events such as headache, somnolence and fatigue.

Bilastine was reported to be safe and well-tolerated even after 1 year of treatment and was recommended as one of the preferred prescriptions for AR treatment (Bousquet et al., 2012). The present meta-analysis accentuates this recommendation. Bilastine showed an overall favorable effect on relieving the symptoms of AR compared to placebo as measured by total symptom score, nasal symptom score, and non-nasal symptom score despite heterogeneity among individual trials. There was no difference in terms of efficacy between bilastine and other OAHs such as cetirizine, desloratadine and fexofenadine for the primary outcomes. Bilastine improved the quality of life of patients compared to placebo for the limited number of trials included. Both bilastine and desloratadine were equally effective in improving the quality of life of patients.

The heterogeneity among the trials is contributed by the different type of AR assessed. Two of the included trials (Bachert et al., 2009; Kuna et al., 2009) recruited patients having seasonal AR while the other two evaluated patients with perennial AR (Sastre et al., 2012; Okubo et al., 2017). Notable discrepancies were found in the scoring of the total symptoms score and severity of symptoms among the trials. In the assessment for the total symptom score, one trial (Bachert et al., 2009) included four nasal and six non-nasal symptoms, one trial (Kuna et al., 2009) included four nasal and three non-nasal symptoms (ocular itching, redness and tearing), one trial (Okubo et al., 2017) included four nasal symptoms and one trial (Sastre et al., 2012) included four nasal and two non-nasal symptoms (ocular redness and tearing). In the assessment for the severity of symptoms, three trials (Bachert et al., 2009; Kuna et al., 2009; Sastre et al., 2012) evaluated the nasal and ocular symptoms on a four-point Likert scale, while one trial (Okubo et al., 2017) evaluated the nasal symptoms using a five-point scale.

Although, the present meta-analysis found bilastine was equal to other OAHs in terms of efficacy there were distinct differences which could be used to select the ideal agent. Bilastine has a rapid onset of action, approximately 1 h after ingestion with sustained duration of action of more than 26 h (Horak et al., 2010). While cetirizine 10 mg has almost a similar profile, fexofenadine 120 mg was only effective up to first 4 h after dosing. Thereafter, bilastine was significantly more effective than fexofenadine in reducing the symptoms of AR. Another consideration is the food-drug interaction. It is common knowledge that the absorption of OAHs might be altered by consumption of certain foods (Bousquet et al., 2012). The bioavailability of bilastine is reduced by approximately 30 % when ingested together with food and grapefruit juice. Thus, to avoid such food-drug interaction, it is recommended that bilastine should be taken 1 h before or 2 h after food or fruit juice intake.

Lynde et al. (2020) observed that the use of first generation OAHs was associated with significant adverse effects namely sedation that negatively impacted the quality of life of patients. With the use of bilastine, a non-sedating OAH, the sedative effect of the older generation OAHs is circumvented. In a meta-analysis by Snidvongs et al. (2017), levocetirizine and cetirizine were acknowledged as having sedative effects. Correspondingly, our meta-analysis showed cetirizine caused greater somnolence than bilastine. The results of the present meta-analysis suggest that even though both bilastine and cetirizine are equally effective, those who require concentration and attentiveness in their works such as handling machinery, drivers or pilots, should be prescribed a non-sedating OAH to avoid this potential adverse effect. Interestingly, a study investigated the use of bilastine under more extreme condition that requires high reactivity and alertness by conducting driving test at Formula One (F1)-high speed simulator where capability to maintain central position and constant speed was measured (Demonte et al., 2018). The study found treatment with bilastine 20 mg on initiation and across the duration of treatment, did not impair the ability of the drivers in adhering to the measured parameters.

### 4.2 Strengths and Limitations

The strength of this review is the assessment of the nasal symptom, non-nasal symptom, quality of life and adverse events. The limitations of this study include the different study protocol between each trial, no comparison of dosing between 10 versus 20 mg daily and no assessment of safety in the elderly patients. In addition, the short treatment period of 2 weeks reported by most of the trials, which may not resemble the actual real-world data. Bilastine was shown to be safe for use in children as young as 2 years of age and it has been approved for use in Europe for children age 6–12 years of age (Novak et al., 2016; Lynde et al., 2020). But due to the relatively meagre data, further investigations are advocated to assess the efficacy and safety of bilastine in children. More investigations are also required to evaluate the efficacy and safety of bilastine with other pharmacotherapy for AR such as intranasal corticosteroid spray and leukotriene receptor antagonist.

## 5 Conclusions

This meta-analysis found bilastine is efficacious and safe in treating AR based on moderate to high quality evidence. Bilastine is effective and safe in treating the overall symptoms of AR with comparable efficacy and safety with other OAHs except somnolence. Whilst bilastine has similar efficacy to cetirizine, somnolence is notably less in bilastine.(Church and Church, 2013).
