# Supplementary material for: Efficacy and Safety of Bilastine in the Treatment of Allergic Rhinitis: A Systematic Review and Meta-analysis
Source: Front Pharmacol. 2022 Jan 10;12:731201. doi: 10.3389/fphar.2021.731201 (PMC8784885; doi:10.3389/fphar.2021.731201)
Supplement: Supplementary file 1 [file DataSheet1.PDF]

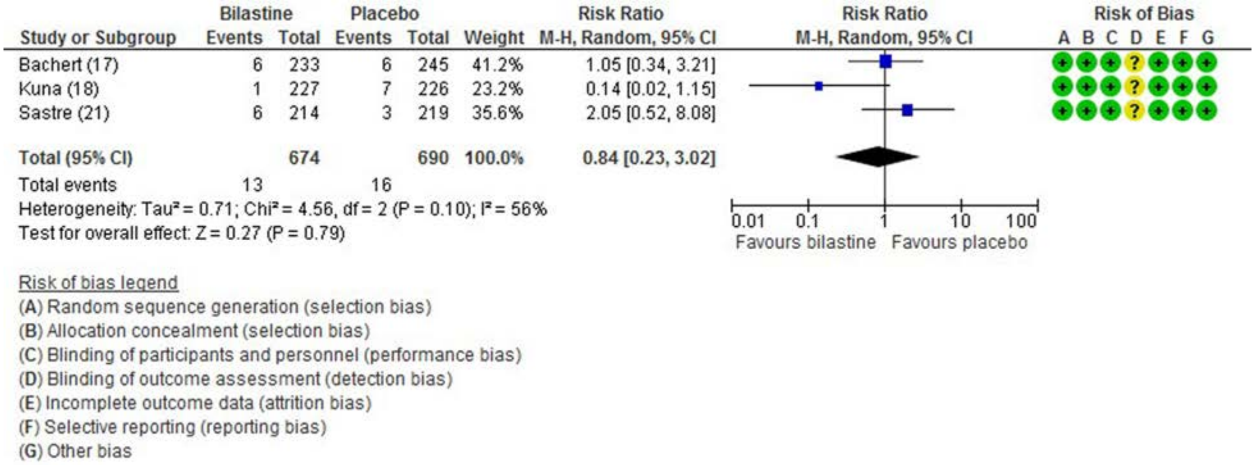

Supplementary Figure 1. Comparison between bilastine and placebo for fatigue. *CI* Confidence interval, *df* degrees of freedom

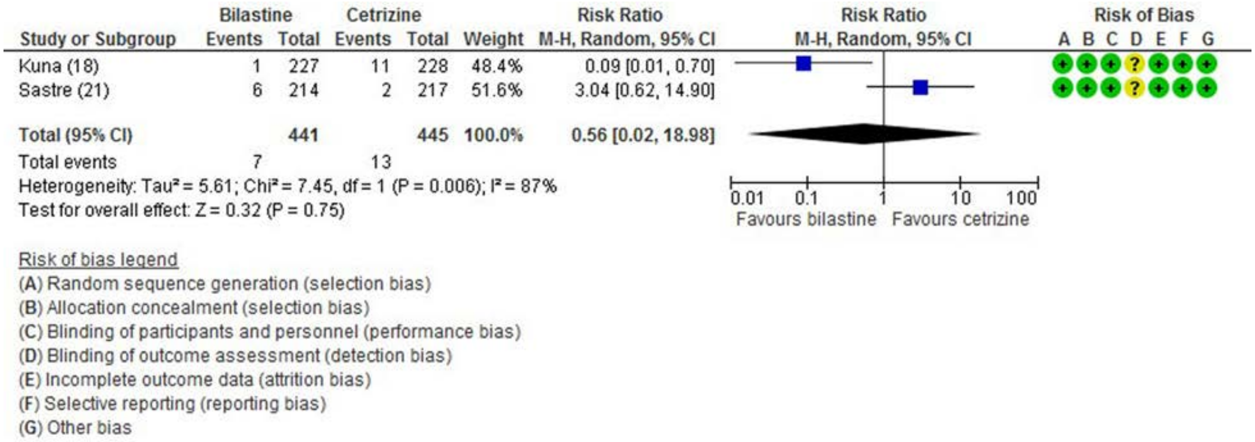

Supplementary Figure 2. Comparison between bilastine and cetirizine for fatigue. *CI* Confidence interval, *df* degrees of freedom
